# Transcriptional landscape of epithelial and immune cell populations revealed through FACS-seq of healthy human skin

**DOI:** 10.1038/s41598-017-01468-y

**Published:** 2017-05-02

**Authors:** Richard S. Ahn, Keyon Taravati, Kevin Lai, Kristina M. Lee, Joanne Nititham, Rashmi Gupta, David S. Chang, Sarah T. Arron, Michael Rosenblum, Wilson Liao

**Affiliations:** 1 Department of Dermatology, University of California, San Francisco, San Francisco, CA United States; 2 Department of Plastic Surgery, California Pacific Medical Center, San Francisco, CA United States; 3 Department of Surgery, University of California, San Francisco, San Francisco, CA United States

**Keywords:** Translational research, Molecular medicine

## Abstract

Human skin consists of multiple cell types, including epithelial, immune, and stromal cells. Transcriptomic analyses have previously been performed from bulk skin samples or from epithelial and immune cells expanded in cell culture. However, transcriptomic analysis of bulk skin tends to drown out expression signals from relatively rare cells while cell culture methods may significantly alter cellular phenotypes and gene expression profiles. To identify distinct transcriptomic profiles of multiple cell populations without substantially altering cell phenotypes, we employed a fluorescence activated cell sorting method to isolate keratinocytes, dendritic cells, CD4+ T effector cells, and CD8+ T effector cells from healthy skin samples, followed by RNA-seq of each cell population. Principal components analysis revealed distinct clustering of cell types across samples, while differential expression and coexpression network analyses revealed transcriptional profiles of individual cell populations distinct from bulk skin, most strikingly in the least abundant CD8+ T effector population. Our work provides a high resolution view of cutaneous cellular gene expression and suggests that transcriptomic profiling of bulk skin may inadequately capture the contribution of less abundant cell types.

## Introduction

As an alternative to microarray-based transcriptomic studies, cDNA-based RNA-seq technology has been widely adopted to characterize the transcriptional landscape of whole human skin tissue and to identify de novo transcripts. For example, in the skin disorder psoriasis, RNA-seq has enabled researchers to identify thousands of differentially expressed coding genes^1^ and long non-coding RNAs^2^ between lesional skin samples taken from psoriasis patients and healthy skin samples taken from healthy controls. However, as whole human skin consists of a heterogeneous mixture of epithelial, immune, and stromal cells, such studies do not provide a clear picture of the cell types in which the differential expression is occurring. More broadly. RNA-seq data derived from complex tissue makes it difficult to understand the biology and associated pathways of individual cell types. Moreover, there is a question as to whether RNA-seq of whole skin fully captures gene expression signals from relatively low abundance and non-uniformly distributed cells such as immune cell subpopulations. This problem can be partially resolved by implementing cell culture methods to grow purified cell populations and performing transcriptome analysis on these purified populations^3–6^. However, cell culture methods may significantly alter cellular phenotypes and gene expression profiles. Recently, high-throughput single-cell RNA-seq (scRNA-seq) based on high-throughput microfluidic capture of cells^7–9^, has provided a way to enrich for low abundance cell types and detect previously undetectable gene expression signals. However, as scRNA-seq is still a relatively new and expensive technology, with several methodological hurdles to overcome, especially with regards to high-throughput single-cell capture, library preparation, and computation^10, 11^.

A protocol for fluorescence-activated cell sorting (FACS) of cell populations followed by RNA-seq (FACS-seq) has recently been described^12^ in sorted macrophage populations from zebrafish larvae. This general approach provides an attractive alternative to scRNA-seq. FACS-seq allows for detection of relatively rarer cell type-specific transcripts at a much lower cost than scRNA-seq, both in terms of money and time because cells do not have to be captured into single wells or capture sites via microfluidics or laser micro-dissection. Furthermore, after cell populations have been sorted, library prep for FACS-seq is identical to library prep for bulk RNA-seq and does not require bar-coding or reagents that have been developed specifically for scRNA-seq. In this study, we implemented FACS-seq to sort out and sequence populations of keratinocytes, dendritic cells, CD4+ T cells, and CD8+ T cells taken from healthy human skin samples. We asked if there are cell type-specific DE genes, gene networks associated with each cell type, and if cell type-specific signature genes are well characterized in bulk samples. Here, we demonstrate the advantages of FACS-seq over traditional bulk RNA-seq, particularly with relatively less abundant cell types such as CD8+ T cells.

## Results

**Figure 1:**
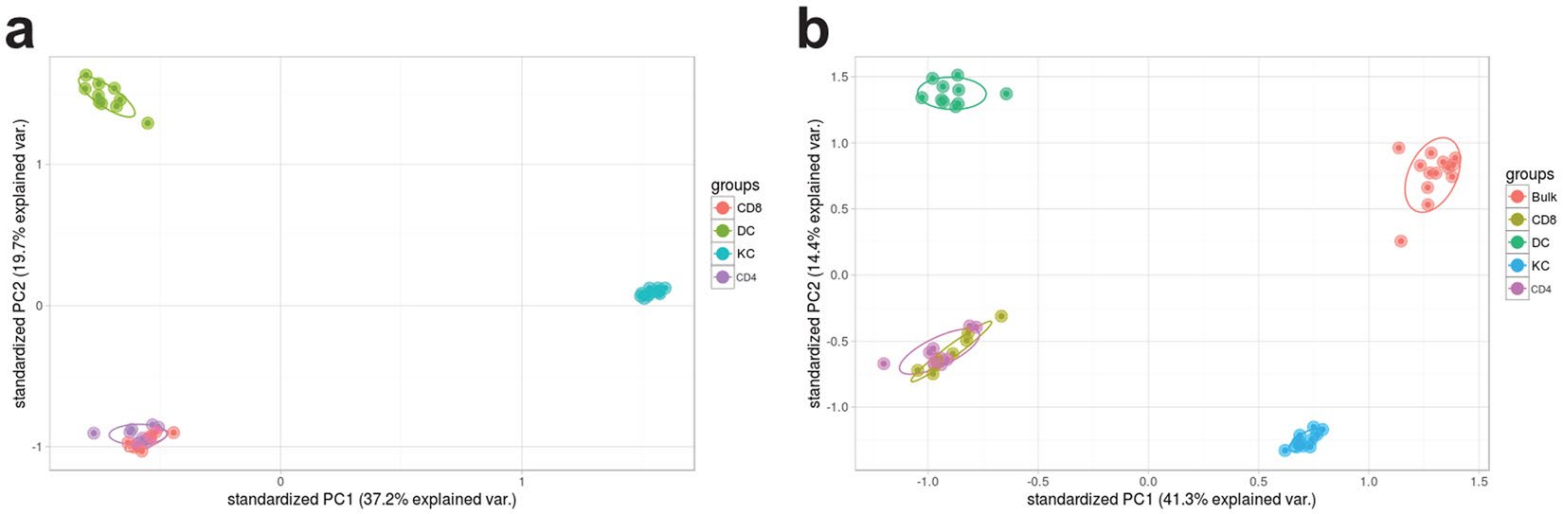
Principal components analysis (PCA) reveals cell populations form distinct clusters. (**a**) First two principal components from PCA of keratinocyte, dendritic cell, CD4+ T cell, and CD8+ T cell populations. (**b**) First two principal components from PCA of populations from (**a**) along with bulk RNA-seq samples.

**Figure 2:**
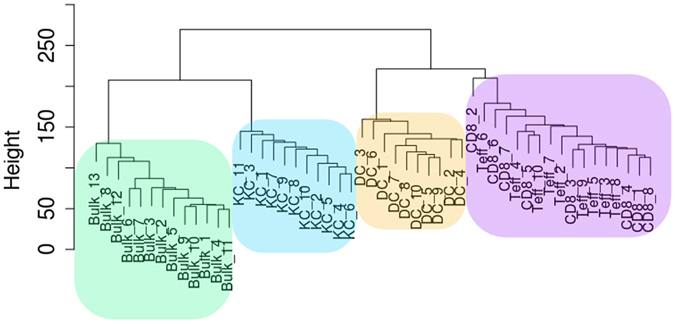
Unsupervised hierarchical clustering reveals that the keratinocyte populations are most similar to bulk samples. Dendrogram from unsupervised hierarchical clustering of each cell population and the bulk samples.

We sorted out purified populations of keratinocytes, dendritic cells, CD4+ T cells, and CD8+ T cells from whole skin obtained from 11 healthy individuals. From these populations, we were able to successfully sequence the transcriptomes of all 11 keratinocyte populations, 10/11 dendritic cell populations, 10/11 CD4+ T cell populations, and 8/11 CD8+ T cell populations. Detailed cell counts from each population are in Supplementary Table S1. Principal components analysis (PCA) of normalized expression revealed that the keratinocytes, dendritic cells, and T cells (CD4+ and CD8+ ) form distinct clusters (Fig. 1a). As the T cells were non-activated, the CD4+ T cells and CD8+ T cells did not further segregate into distinct clusters. We also performed RNA-seq on full thickness human skin (see Methods). When these bulk control samples were included in the PCA, distinct clusters were observed in each quadrant of the plot, with the bulk control samples and keratinocytes sharing the same horizontal axis (PC1, comprising the largest fraction of the sample variance) and the dendritic cells and T cells also sharing the same horizontal axis, suggesting that keratinocytes are transcriptomically most similar to the bulk samples (Fig. 1b). Hierarchical clustering also showed that the bulk control samples were most similar to the keratinocytes (Fig. 2). The dendrogram showed two major branches, with the bulk controls and the keratinocytes forming sub-branches on one of the major branches and the dendritic cells and the T cells forming sub-branches on the other major branch.

### Identifying Signatures of Epithelial and Immune Cells

#### DE between populations

**Figure 3:**
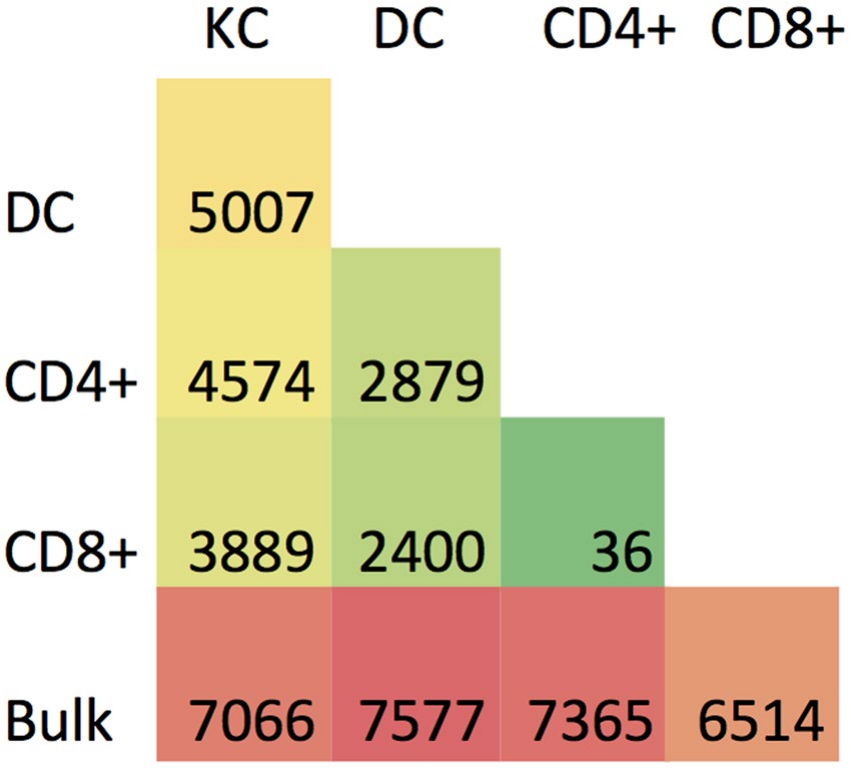
Pairwise differential expression analysis between cell populations reveals greatest difference between each cell population and bulk samples. Pairwise differential expression analysis was performed between each cell population and the bulk samples and between each cell population. Green coloring indicates fewer differentially expressed genes while red coloring indicates the most differentially expressed genes in a pair.

To gain insight into the biology of native cell populations within human skin, we performed pairwise differential expression testing between each cell population and bulk samples as well as between each of the cell populations (Fig. 3). Keratinocytes showed the greatest differential expression in comparisons with all other cell types, with the number of differentially expressed genes ranging between 3889 and 5007 (q ≤ 0.05). Not surprisingly, the CD4+ T effectors differed the least from the CD8+ T effectors with only 36 differentially expressed genes between the two populations (q ≤ 0.05) (See Supplementary Table S2 for all DE genes).

### KC vs Bulk

7066 genes were differentially expressed (q ≤ 0.05) between the bulk samples and the keratinocyte samples, with 3159 genes over-expressed in the keratinocyte samples (See Supplementary Table S2 for all DE genes). The most up-regulated gene was *DLK1* (log_2_FC = 6.97, q = 0.02), while the most down-regulated gene was *COL3A1* (log_2_FC = −15.86, q = 7.5E-4). The top 100 up-regulated genes in the keratinocytes were enriched for Gene Ontology (GO) biological process (BP) terms that included “mammary gland epithelium development” (p = 2.70 × 10^−3^), “epithelial cell differentiation” (p = 0.013), and “epithelium development” (p = 0.017) (see Supplementary Table S3 for full list of GO BP terms). From a list of 76 keratinocyte specific genes^3^, 40 were in common with the 7066 DE genes, including *KRT6A*, *KRT16*, *LGALS7*, and *S100A8*.

### DC vs Bulk

We found 7577 genes were differentially expressed (q ≤ 0.05) between the bulk samples and the dendritic cell samples, with 2843 genes over-expressed in the dendritic cell samples (See Supplementary Table S2 for all DE genes). Professional antigen presenting cell (APC) specific genes such as *CIITA*, *CD40*, *CD80*, and *CD86* were found to be amongst the over-expressed genes. Top GO BP terms enriched for amongst the top 100 up-regulated genes in the dendritic cells included “response to lipopolysaccharide” (p = 2.60 × 10^−24^), “response to molecule of bacterial origin” (p = 6.69 × 10^−24^), and “inflammatory response” (p = 7.49 × 10^−23^) (see Supplementary Table S3 for full list of GO BP terms). A recent study by Polak *et al*.^13^ identified 744 genes that were overexpressed in unstimulated dermal dendritic cells (dDCs) and 519 genes overexpressed in unstimulated Langerhans Cells (LCs). Of the 2843 over-expressed genes, 363 were in common with the 744 dDC genes and 45 were in common with the 519 LC genes suggesting that while both LCs and dDCs were present in the epidermis of the samples, dDCs make up a larger proportion of the dendritic cells present.

### CD4+ T cell vs Bulk

We found 7365 genes were differentially expressed (q ≤ 0.05) between the bulk samples and the CD4+ T cells, with 2643 over-expressed in the CD4+ T cell samples (See Supplementary Table S2 for all DE genes). These over-expressed genes were enriched for GO biological process terms such as “leukocyte activation” (p = 1.72 × 10^−25^), “lymphocyte activation” (p = 3.05 × 10^−25^), and “cell activation” (p = 1.29 × 10^−22^) (see Supplementary Table S3 for full list of GO BP terms). Palmer *et al*.^14^ identified 23 T cell signature genes and of these 23 genes, 18 were in common with the genes over-expressed amongst the CD4+ T cells, including *CD3G*, *CD3D*, *CD28*, and *LEF1*.

### CD8+ T cells vs Bulk

6514 genes were differentially expressed between the bulk samples and the CD8+ T cell population, with 2659 being over-expressed in the CD8+ T cell population (See Supplementary Table S2 for all DE genes). Significantly enriched GO biological process terms included “leukocyte activation” (p = 1.69 × 10^−29^), “lymphocyte activation” (p = 3.72 × 10^−28^) (see Supplementary Table S3 for full list of GO BP terms). Of the 12 genes that Palmer *et al*.^14^ identified as CD8+ T cell specific, 9 were included with the genes that were over-expressed amongst the CD8+ T cells, including *CD8A*, *CD8B*, *CCL5*, *PRF1*, and *GZMH*.

### Network analysis reveals population specific networks

**Figure 4:**
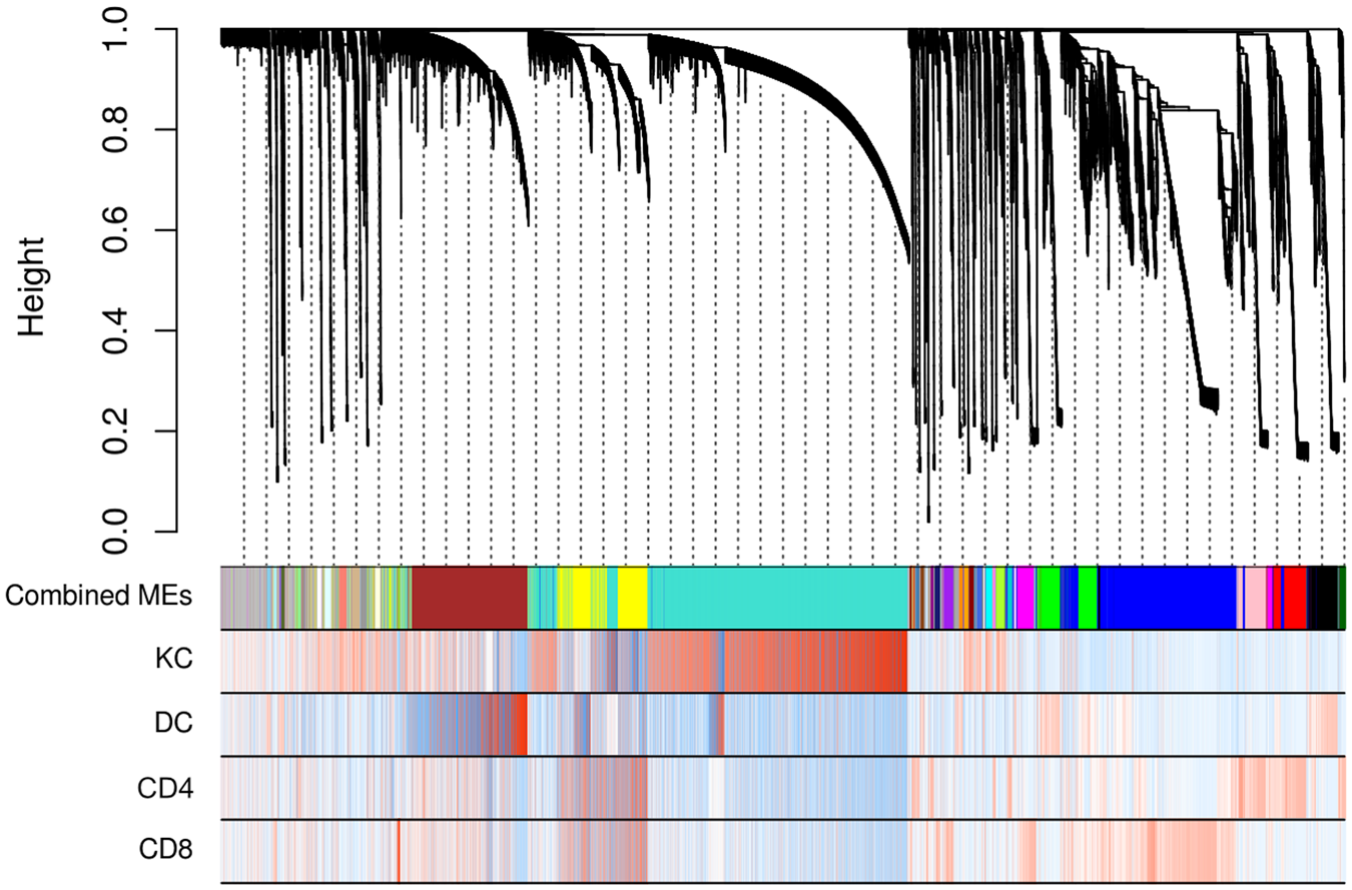
Cluster dendrogram reveals modules that were positively correlated with a specific cell type. Hierarchical clustering dendrogram of modules identified by WGCNA with branches corresponding to module color assignments in the first color band beneath the dendrogram. The remaining color bands show how positively (red) or negatively (blue) an individual gene is correlated with a specific module and cell type.

While differential expression analysis revealed genes that were differentially expressed between populations and the bulk samples on an individual gene basis, we wanted to know if there were also cell type-specific networks of genes. Towards this end, we implemented weighted gene coexpression network analysis (WGCNA)^15^ on the entire set of FACS-sorted cell types. WGCNA is an unsupervised and unbiased method to identify networks of coexpressed genes (or modules) followed by calculation of module eigengenes (ME), which is the first principal component of each module. Each ME was then correlated with a cell type-specific phenotype. Genes were assigned to modules and modules were subsequently merged based on their similarity, with modules showing pairwise correlation of 0.7 being merged. Figure 4 depicts a cluster dendrogram showing module assignment as well as a heat map showing how positively (red) or negatively (blue) an individual gene is correlated with a specific cell type. We detected 43 coexpression networks or modules which were randomly labeled with colors.

**Figure 5:**
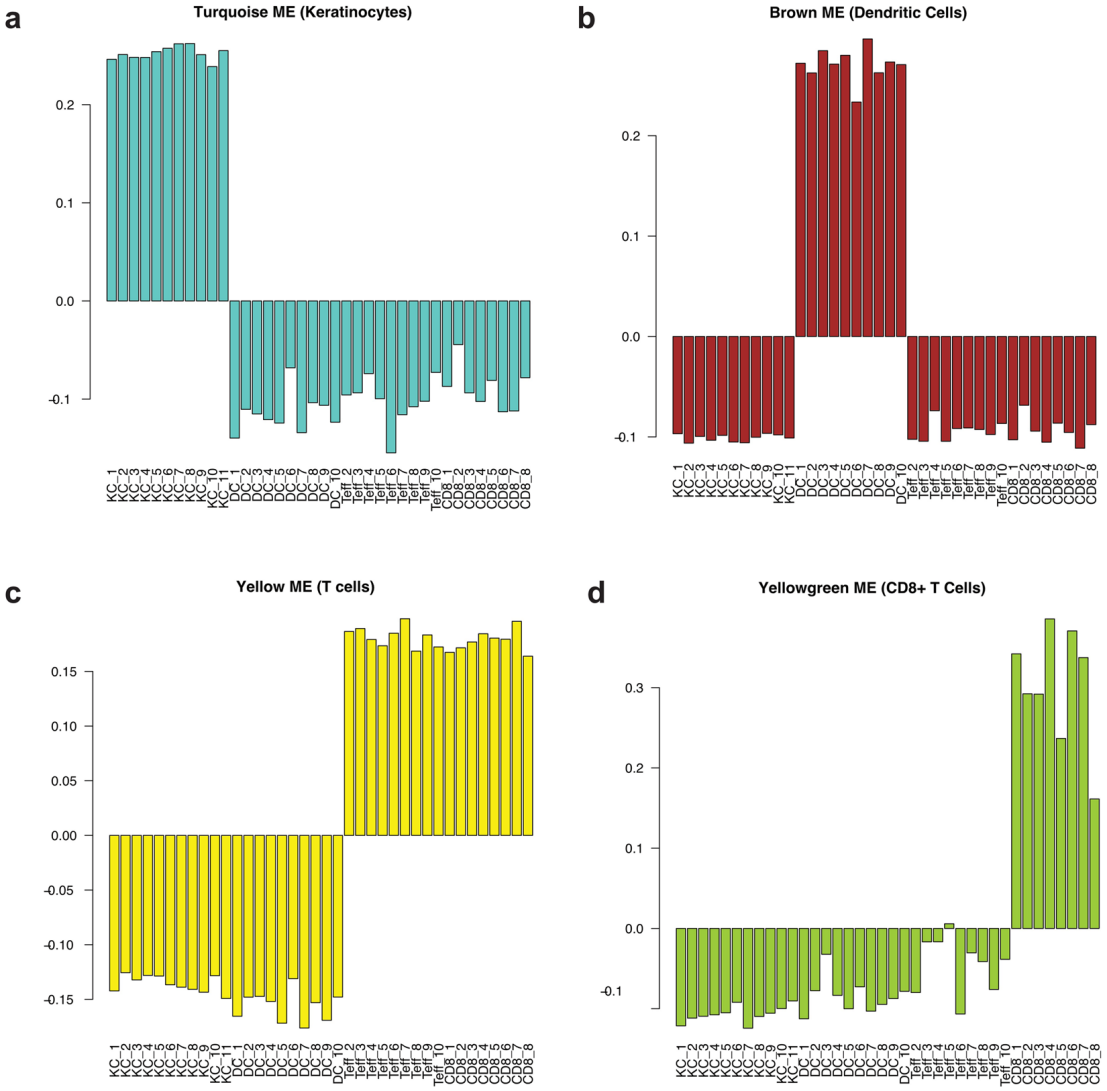
Barplots of the module eigengene (ME) reveal distinct patterns of ME overexpression for each cell type. Barplots of ME expression across all samples for modules that are associated with keratinocytes (**a**), dendritic cells (**b**), T cells (**c**), and CD8+ T cells (*d*).

**Figure 6:**
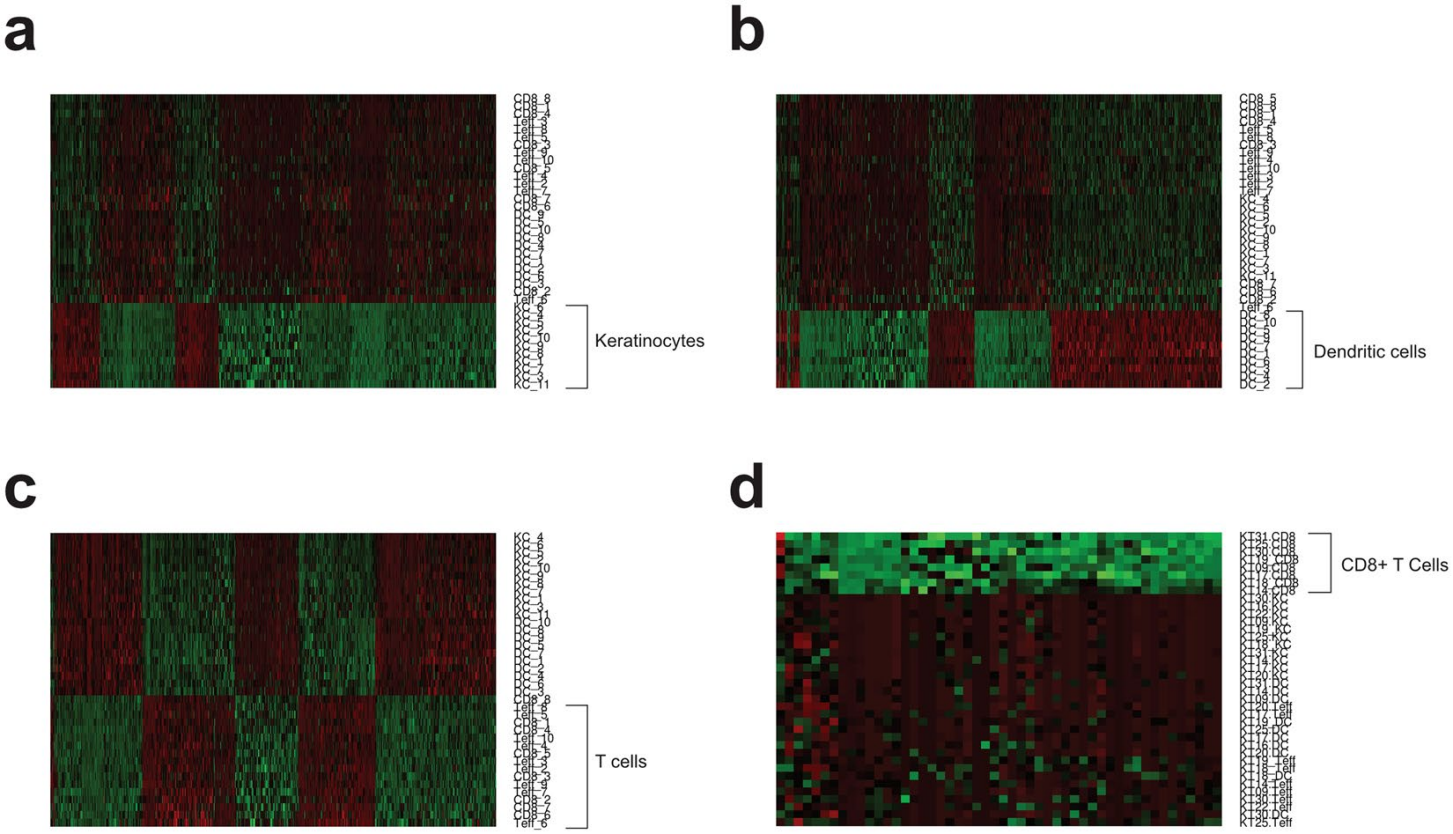
Gene expression heatmapping of module genes reveals cell type-specific patterns of expression. Gene expression heatmap of all module genes for the keratinocyte associated module (**a**), the dendritic cell associated module (**b**), the T cell associated module (**c**), and the CD8+ T cell associated module (**d**).

The module eigengene (ME) is the first principal component of a module and can be viewed as a representative for the overall expression profile of a module. To test if a module is associated with a particular cell type, we correlated the MEs of all 43 modules with the phenotypes of each cell type. The turquoise, brown, and yellowgreen MEs were significantly correlated with keratinocytes, dendritic cells, and CD8+ T cells, respectively (FDR ≤0.05 and Pearson correlation coefficient ≥0.95). A fourth ME (yellow) was significantly correlated with T cells (not specific for CD4+ or CD8+ T cells) with a FDR ≤0.05 and correlation coefficient of 0.62 (Table 1). Barplots of each ME across all samples also revealed that distinct patterns of overexpression in each of the four significantly correlated modules (Fig. 5a–d). Heatmaps combined with hierarchical clustering of the turquoise, brown, yellow, and yellow green module genes also revealed distinct patterns of individual gene expression specific to keratinocytes, dendritic cells, T cells, and CD8+ T cells (Fig. 6a–d).

**Table 1:** Module eigengenes that are significantly correlated with each cell type (FDR ≤ 0.05).

Cell Type	Module	Trait Correlation	FDR_Trait Correlation_	#Of genes	#Overexpressed
Keratinocyte	turquoise	0.99	1.15 × 10^−32^	6247	1748
Dendritic cell	brown	0.99	1.87 × 10^−41^	2472	892
CD4+ T cell	yellow	0.62	1.21 × 10^−3^	1745	725
CD8+ T cell	yellowgreen	0.96	2.81 × 10^−20^	50	26

Within each module, we wanted to know which genes were the most influential. To identify these “hub” genes, we calculated the module membership (MM) for each gene as well as an individual gene significance measure. Using a threshold of MM ≥ 0.9 and a p_gene significance_ ≤ 5 × 10^−4^, we identified 760 hub genes in the keratinocyte module (n_genes_ = 6427), 266 in the dendritic cell module (n_genes_ = 2472), 155 in the T cell module (n_genes_ = 1745), and 17 in the CD8+ T cell module (n_genes_ = 50).

Top keratinocyte module hub genes included *SAMSN1*, *ESRP1*, and *MALAT1* and top GO BP terms that were enriched for included “epithelium development” (p = 2.35 × 10^−25^), “epidermis development” (p = 1.79 × 10^−24^), and “skin development” (p = 9.77 × 10^−21^). Top dendritic cell module hub genes included *SLC7A11*, *PAPSS2*, *KYNU*, and *BMP6*, while top GO BP terms that were enriched for included “immune response” (p = 6.95 × 10^−22^), “defense response” (p = 1.51 × 10^−20^), and “inflammatory response” (p = 5.89 × 10^−17^). For the T cell module, top hub genes included *THEMIS*, *CD3E*, and *CD2*, with enrichment for GO BP terms including “homotypic cell-cell adhesion” (p = 2.79 × 10^−13^), “T cell aggregation” (p = 4.70 × 10^−13^), and “T cell activation” (p = 4.70 × 10^−13^). Finally, for the CD8+ T cell module, top hub genes included *KLRC3*, *SAMD3*, *CRTAM*, and *KLRK1*, with the top hub genes enriching for GO BP terms such as “immune effector process” (p = 3.62 × 10^−8^), “defense response” (p = 8.01 × 10^−8^), and “lymphocyte activation” (p = 2.03 × 10^−7^) (see Supplementary Table S4 for all module hub genes and Supplementary Table S5 for all top hub gene GO BP terms).

**Figure 7:**
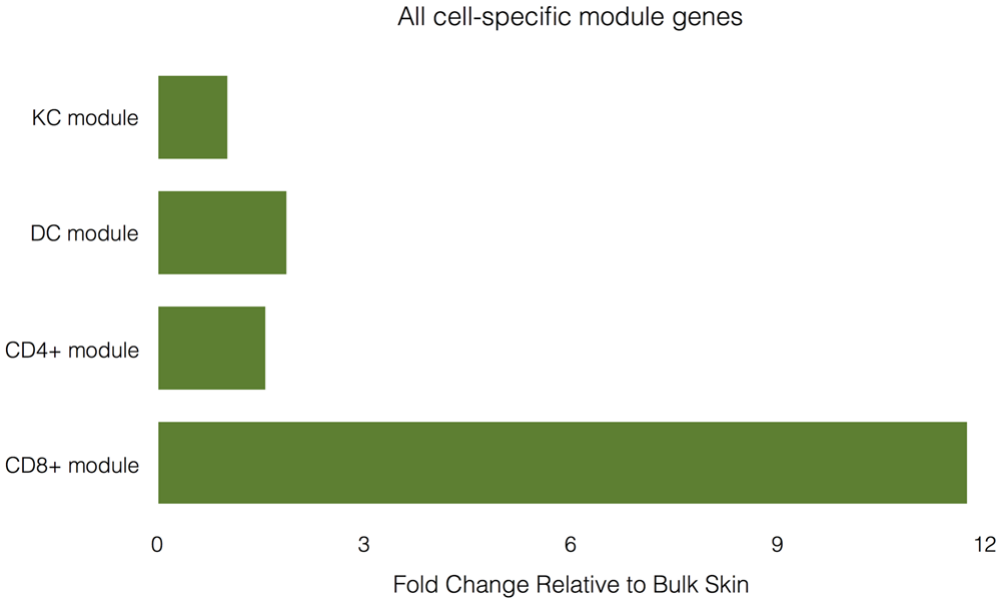
Most highly expressed genes from the CD8+ T cell module were enriched for nearly 12-fold in CD8+ T cell populations relative to bulk samples. The summed expression of the top 50 most expressed genes from each module was averaged across all samples in each cell population and from the bulk samples. Fold change relative to bulk skin is defined as the ratio of the average summed expression across samples in each population to the average summed expression in the bulk samples.

When we calculated the summed expression of the top 50 most highly expressed genes from each module across each sample, CD8+ module genes were enriched for in the CD8+ population samples versus the bulk samples by nearly 12-fold on average (Fig. 7). In contrast, the top 50 genes from the T cell, dendritic cell, and keratinocyte modules were only enriched by 3.95, 3.3, and 2.49, respectively. Furthermore, CD8+ module genes that were of low-abundance (FPKM < 1) in bulk skin such as *CRTAM* (mean FPKM_bulk_ = 0.03) and *NKG7* (mean FPKM_bulk_ = 0.01) had over 1000-fold higher expression in the CD8+ populations (*CRTAM* mean FPKM_CD8_ = 51.72, *NKG7* mean FPKM_CD8_ = 147.64). As CD4+ T cells, dendritic cells, and keratinocytes are more abundant in the skin than CD8+ T cells, this again suggests that CD8+ T cells are not well represented in the bulk samples.

## Discussion

In this study, we implemented FACS-seq on whole skin biopsies of healthy skin to determine cell type-specific DE genes, gene coexpression networks correlated with a particular cell type, and if cell type-specific signature genes are well represented in bulk RNA-seq. Our results show that there are thousands of DE genes between cell populations (with the exception between CD4+ T effectors and CD8+ T effectors) and between each population and bulk skin. We also show that each cell population is correlated with a specific coexpression network module and that the hub genes within each module may not be adequately represented in the bulk samples, particularly the CD8+ T effector population.

FACS and RNA-seq are now mature technologies with standardized hardware and software protocols and routinely implemented by labs worldwide. Combining the two technologies does not require any new technologies, reagents, or specialized protocols to yield cell type-specific results. This is in sharp contrast to scRNA-seq that requires specialized protocols for capturing single-cells as well as specialized, expensive reagents that were developed specifically for scRNA-seq. FACS-seq also does not require specialized downstream computational tools. For instance, to perform differential expression analysis on single-cell data, newly-developed computational tools that can properly model the variance in gene expression between single-cells must be applied. With FACS-seq, tools developed for analyzing bulk RNA-seq data such as the EdgeR^16^ and Cufflinks suite^17^ can be implemented.

Methods such as CIBERSORT^18^ now allow for computational deconvolution of cell types from bulk tissue expression data. However, these methods are not yet generalizable to all tissue or cell types—particularly resident cell types in the skin—and the uncertainty of the inferred types is not yet negligible. FACS-seq allows for confident assignment of biological function of genes to specific cell types without having to first computationally infer the cell type from the expression data. While FACS-seq may be marginally more expensive than bulk RNA-seq followed by computational deconvolution, this increased cost is offset by the increased confidence in cell type-specific differential expression or network analysis.

We have shown that cell type-specific gene expression from relatively rarer cell types in the skin, may be drowned out in bulk samples. For instance, CD8+ T cell signature genes are expressed several-fold higher in the CD8+ T cell populations over the bulk samples. This may be due to the presence of fewer T cells in the skin relative to keratinocytes or dendritic cells.

In bulk RNA-seq, library preparation reagents are more likely to amplify transcript fragments from relatively higher abundance cell types in skin such as keratinocytes. Even with sample biopsies of the same dimensions, we can avoid the transcript amplification bias of higher abundance cell types with FACS-seq because library preparation occurs independently for each cell population that is sorted.

Under bulk RNA-seq some disease associated SNPs (causal SNPs) at eQTLs may not be correlated with a gene expression change because because the change in gene expression levels may only affect certain cell types. Westra *et al*.^19^ showed that it is possible to determine the effect of SNPs at eQTLs using bulk tissue expression data by computationally inferring the cell types. However, computational deconvolution may not capture relatively rare expression signals that are only picked up when cell populations are sorted and purified.

While we did detect a T cell specific module and a CD8+ T cell specific module, we were not able to detect a CD4+ T cell specific module. One explanation, from Palmer *et al*.^14^ is that CD4+ and CD8+ T cells both have a common transcriptomic core prior to stimulation and while CD8+ T cells express some cytotoxic genes prior to stimulation, CD4+ T cells may only express specific genes when stimulated. Perhaps another explanation is the lower than expected abundance of CD4+ T cells relative to CD8+ T cells. We also note that the dendrogram plot shows that resting CD8+ T cells and CD4+ T cells share much in common, which is bolstered by the fact that only 36 genes are DE between them.

As observed by Palmer *et al*.^14^, unactivated/resting CD8+ T cells distinguish themselves by signature genes encoding cell surface receptors and cytotoxic function. Many of these genes identified in Palmer *et al*. that are CD8+ specific are in the CD8+ module. We may infer from these observations that even in a resting state, CD8+ T cells come with “batteries loaded”.

There were several limitations of the present study. Human skin is composed of a large number of cell types including fibroblasts, adipocytes, melanocytes, and vascular cells, however here we focused on FACS-seq of keratinocytes, dendritic cells, and T effectors. We also did not match each individual sample’s cell populations with their respective bulk skin sample. However, as all samples came from healthy individuals, we do not believe that there would be significant differences in the transcriptional landscape of the bulk samples across individuals. We note that technical limitations of FACS-seq include a requirement that tissue samples must be fresh (only refrigerated, not frozen), access to qualified technicians that can perform the tissue digestion, potential confounding due to overnight tissue processing (which we controlled for by only including samples with RNA integrity number (RIN) ≥ 7), and of course, access to a FACS machine. We also note biological limitations due to the variability of immune cell populations residing in human skin that we have shown may be dependent upon factors such as anatomic location^20^, as well as gender and age.

In summary, we have shown for the first time that FACS-seq can provide a clearer picture of the complex transcriptional landscape of human skin. Through differential expression analysis and coexpression network analysis, we show biological processes and pathways that may be associated with individual cell types. Moreover, our study demonstrates that FACS-seq is a cost-effective method to explore the cellular heterogeneity of skin and characterize the transcriptomic signatures of less abundant—but no less interesting—cell types.

## Methods

### Sample collection

Written informed consent for donation of biospecimens was obtained from all subjects and all experimental protocols were approved by the UCSF Institutional Review Board. All methods were carried out in accordance with relevant guidelines and regulations. Surgical skin discard samples from the abdomen, breast, chin, and right lateral chest wall were obtained for 11 adult subjects and processed at the University of California San Francisco (UCSF). Among the 11 subjects, 5 were female and the mean age was 43.2 years (standard deviation = 13.75 years) (See Supplementary Table S6). Skin samples were first processed with a dermatome to remove the top 0.4 mm of the sample. A 4 cm^2^ piece was floated on 5 ml of dispase overnight at 4 C for the keratinocytes. The rest of the tissue was finely minced with scissors and placed in 3 ml of digestion mix (10 ml resting media, 8 mg type IV collagenase (Worthington, Cat# LS004186), and 0.2 mg DNAse (Sigma, Cat# DN251G)) and put into a 37 C incubator overnight (~14 hours). The tissue was washed the next morning with 3 ml wash buffer (500 ml RPMI-1640 medium, 10 ml 2% fetal bovine serum (FBS), 5 ml 1% penicillin-streptomycin (PS)), filtered through a 100 μm filter, and spun down at 1500 rpm for 5 minutes. This pellet was re-suspended in wash buffer and then counted with a Nucleocounter.

The 4 cm^2^ piece of skin floated on dispase was processed by peeling off the epidermis, placing the epidermis in 5 ml phosphate-buffered saline (PBS) and PS followed by transfer into 5 ml of trypsin (0.5% trypsin-EDTA (Gibco, Cat# 15400-054)) for 10 minutes in a 37 C incubator, washing it with PBS and PS, and then spinning it down and counting the cells. The cells were stained with CD45 and Ghost dye. Before running the cells on the sorter, they were put through a 100 μm filter.

### FACS

To find the populations of interest on the FACS machine, a lymphocyte gate was taken and then doublets were excluded in a following gate. From there, live CD45+ cells were gated, then CD45+ CD3+ cells, and from here CD4+ and CD8+ cells could be observed. From the CD4+ gate, CD25 was plotted vs CD27. CD25−CD27− cells were gated and called T effectors. The dendritic cells were found by making a broad gate and then taking the live CD45+ cells. From here, CD45+ CD3− cells were gated and then CD11c+ HLADR+ cells were gated and called dendritic cells. The keratinocytes were in a separate tube and were gated by plotting CD45 vs Ghost dye and taking the cells that were CD45−Ghost dye− (see Supplementary Figure 1 for gating strategy). The cells were sorted into 1.5 mL Eppendorf tubes that were pre-filled with 500 μl of 2% FBS, spun down, and snap frozen in liquid nitrogen for storage at −80 °C. Reported post-sort purity was greater than 90% for all populations.

### Library preparation and RNA-seq

RNA was extracted from biopsy samples using the Qiagen Allprep DNA/RNA mini kit (Qiagen, Valencia, CA). An Agilent 2100 Bioanalyzer was used to identify samples with RIN ≥ 7. The Ovation RNA-Seq System V2 protocol (NuGEN, San Carlos, CA) was implemented to amplify cDNA from 500 pg of qualified mRNA from each sample. In brief, cDNA amplification was performed using NuGEN’s Ribo-SPIA technology to yield several micrograms of cDNA. The cDNA was then sheared as ~200 bp fragments, end-repaired, followed by A-tailing and adapter ligation reactions. The library was then PCR enriched and purified using the Ampure XP bead size selection method to generate the final product. All libraries were quantified by Caliper and real-time qPCR. The qualified libraries were amplified on cBot to generate clusters on the Illumina flowcell, and sequenced on the Illumina HiSeq 2500 platform, yielding an average of 60.7 million 100-bp paired-end reads per sample.

### Read alignment and differential expression analysis

Read quality was checked using FastQC v. 0.11.4^21^. Reads with adapter contamination were trimmed using Trimmomatic v. 0.32^22^. Reads were aligned to the UCSC hg19 human reference genome using STAR v. 2.4.2a^23^. Gene annotations for 26 K coding genes were obtained from RefSeq^24^. We used the Cufflinks Suite v 2.2.1^25^ to test for differential expression.

### Network analysis

After expression values were normalized to the number of reads per kilobase per million reads, we performed QC on the matrix of normalized expression values to remove any transcripts with either zero variance or a missing value and remove samples that were outliers in an initial unsupervised hierarchical clustering analysis. After QC, a weighted adjacency matrix was created, defined as, A_ij_ = |cor*(x_i_, x_j_)|^β^, where x_i_ and x_j_ are the i^th^ and j^th^ genes, respectively. The soft thresholding power parameter, β, was set to 12 after a sensitivity analysis of scale-free topology was performed. This weighted adjacency matrix was used to generate a topological overlap matrix (TOM) and dendrogram. A dynamic hybrid branch cutting method was implemented on the resulting TOM-based dendrogram to identify module eigengenes (ME). MEs are the first principal components for each gene expression module after a singular value decomposition is performed on the TOM. A cut height of 0.3 was set to merge MEs that have a correlation of 0.7 or greater. A phenotypic cell type-based gene significance measure was defined as GS_i_ = |cor*(x_i_, t)|, where x_i_ is the i^th^ gene and t is the binary indicator variable for cell type. An ME significance was defined as MES_i_ = |cor*(ME_j_, t)|, where ME_j_ is the j^th^ ME. Module membership, MM, for the i^th^ gene was defined as, MM = |cor*(x_i_, ME)|.

*Unless otherwise specified, ‘cor’ refers to Pearson correlation.

## Electronic supplementary material

Supplementary Figure 1

Supplementary Table 1

Supplementary Table 2

Supplementary Table 3

Supplementary Table 4

Supplementary Table 5

Supplementary Table 6
