# Author Correction: Transcriptional landscape of epithelial and immune cell populations revealed through FACS-seq of healthy human skin

**DOI:** 10.1038/s41598-022-05431-4

**Published:** 2022-01-17

**Authors:** Richard S. Ahn, Keyon Taravati, Kevin Lai, Kristina M. Lee, Joanne Nititham, Rashmi Gupta, David S. Chang, Sarah T. Arron, Michael Rosenblum, Wilson Liao

**Affiliations:** 1 Department of Dermatology, University of California, San Francisco, San Francisco, CA USA; 2 Department of Plastic Surgery, California Pacific Medical Center, San Francisco, CA USA; 3 Department of Surgery, University of California, San Francisco, San Francisco, CA USA

Correction to: *Scientific Reports* 10.1038/s41598-017-01468-y, published online 02 May 2017

This Article contains errors where the Data availability statement is not included in the original version of this Article. It should read:

**Data availability**

Transcriptomic data generated for this study have been deposited in NCBI GEO under accession number GSE115898.

