# Supplementary material for: Transcriptional landscape of epithelial and immune cell populations revealed through FACS-seq of healthy human skin
Source: Sci Rep. 2017 May 2;7:1343. doi: 10.1038/s41598-017-01468-y (PMC5430950; doi:10.1038/s41598-017-01468-y)

Supplementary Information for "Transcriptional landscape of epithelial and immune cell populations revealed through FACS-seq of healthy human skin"

Richard S. Ahn[1*], Keyon Taravati[1], Kevin Lai[1], Kristina M. Lee[1], Joanne Nititham[1], Rashmi Gupta[1], David S. Chang[2,3], Sarah T. Arron[1], Michael Rosenblum[1], Wilson Liao[1]

[1]Department of Dermatology, University of California, San Francisco, San Francisco, CA. [2]Department of Plastic Surgery, California Pacific Medical Center, San Francisco, CA

[3]Department of Surgery, University of California, San Francisco, San Francisco, CA

[*]Correspondence should be addressed to Richard Ahn, PhD, Department of Dermatology, University of California, San Francisco, 2340 Sutter Street, Box 0808, San Francisco, CA 94143-0808, USA (email: richard.ahn@ucsf.edu)

Supplementary Figure 1a. FACS gating strategy for sorting out CD4+ T effectors, CD8+ T cells, and dendritic cells.

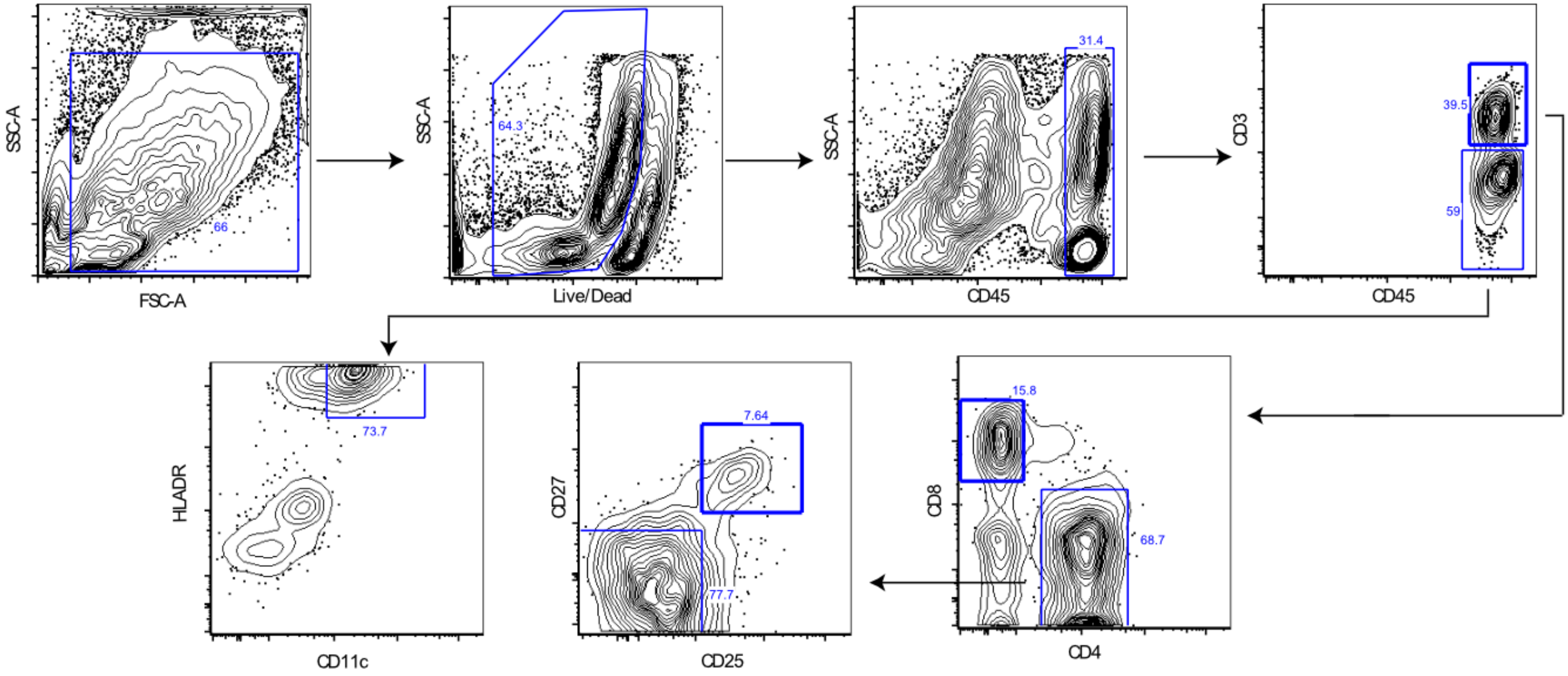

Supplementary Figure 1b. FACS gating strategy for isolating keratinocytes.

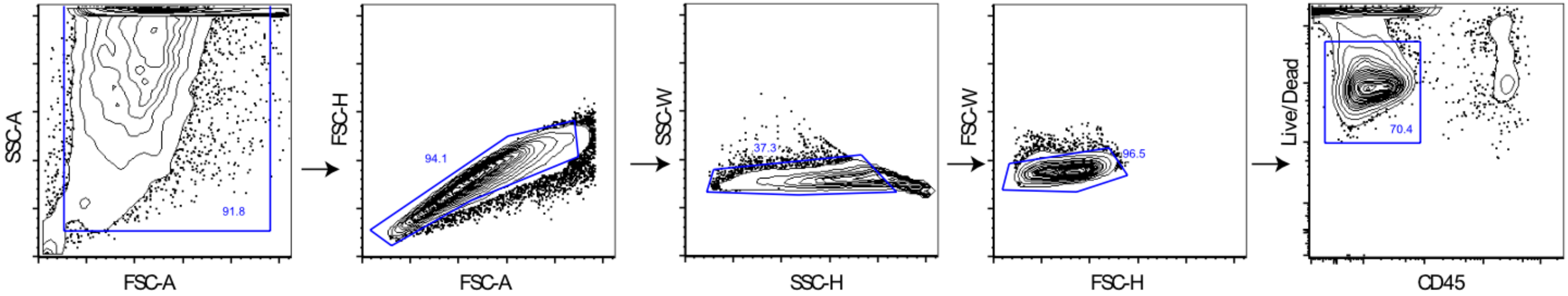

Supplement: Supplementary file 1 — Supplementary Figure 1 [file 41598_2017_1468_MOESM1_ESM.pdf]
